# Rabconnectin-3α/DMXL2 Is Locally Enriched at the Synaptic Ribbon of Rod Photoreceptor Synapses

**DOI:** 10.3390/cells12121665

**Published:** 2023-06-19

**Authors:** Alina Dittrich, Girish Ramesh, Martin Jung, Frank Schmitz

**Affiliations:** 1 Institute of Anatomy and Cell Biology, Saarland University, 66421 Homburg, Germany; alina-dittrich@gmx.de (A.D.); girishr.19@gmail.com (G.R.); 2 Institute of Biophysics, Saarland University, 66421 Homburg, Germany; 3 Institute of Medical Biochemistry and Molecular Biology, Saarland University, 66421 Homburg, Germany; martin.jung@uks.eu

**Keywords:** retina, ribbon synapse, rabconnectin-3α, DMXL2, vesicular H^+^-ATPase, Rab3

## Abstract

Ribbon synapses reliably transmit synaptic signals over a broad signalling range. Rod photoreceptor ribbon synapses are capable of transmitting signals generated by the absorption of single photons. The high precision of ribbon synapses emphasizes the need for particularly efficient signalling mechanisms. Synaptic ribbons are presynaptic specializations of ribbon synapses and are anchored to the active zone. Synaptic ribbons bind many synaptic vesicles that are delivered to the active zone for continuous and faithful signalling. In the present study we demonstrate with independent antibodies at the light- and electron microscopic level that rabconnectin-3α (RC3α)—alternative name Dmx-like 2 (DMXL2)—is localized to the synaptic ribbons of rod photoreceptor synapses in the mouse retina. In the brain, RC3α-containing complexes are known to interact with important components of synaptic vesicles, including Rab3-activating/inactivating enzymes, priming proteins and the vesicular H^+^-ATPase that acidifies the synaptic vesicle lumen to promote full neurotransmitter loading. The association of RC3α/DMXL2 with rod synaptic ribbons of the mouse retina could enable these structures to deliver only fully signalling-competent synaptic vesicles to the active zone thus contributing to reliable synaptic communication.

## 1. Introduction

Ribbon synapses are specialized, continuously active synapses built in the retina, pineal gland and inner ear [1,2,3,4,5,6,7]. In the retina, rod and cone photoreceptors and bipolar cells form ribbon synapses. Ribbon synapses faithfully transmit a broad range of stimulus intensities by computing graded changes of membrane potential. Rod ribbon synapses can even reliably transmit signals generated by the detection of single photons [8,9,10,11,12,13,14,15,16,17,18,19]. The highly reliable synaptic transmission at this type of synapse requires structural and functional specializations. The synaptic ribbon is the characteristic presynaptic structural specialization of ribbon synapses. Synaptic ribbons bind many synaptic vesicles and deliver them to the active zone to promote continuous synaptic transmission in a precise and largely indefatigable manner at high temporal resolution. RIBEYE is the main building block of synaptic ribbons and is essential to make the synaptic ribbon [20,21,22,23,24,25,26]. RIBEYE consists of a unique amino-terminal A-domain. The carboxyterminal B-domain of RIBEYE is identical to CtBP2 except for the first 20 amino-terminal amino acids [20]. CtBP2 functions as an NAD(H)-binding nuclear co-repressor and is highly homologous to CtBP1. The latter proteins evolved from D-isomer-specific 2-hydroxyacid dehydrogenases [27,28,29,30].

Intense synaptic vesicle trafficking events occur at the synaptic ribbon [7,31,32]. Synaptic vesicles bind to the synaptic ribbon and translocate along the ribbon to the active zone at which synaptic vesicle fusion occurs [33,34,35,36,37]. At many synapses, the synaptic vesicle-associated small GTP-binding protein Rab3A [38,39] is important for the recruitment of synaptic vesicles to the active zones, thereby also mediating aspects of synaptic plasticity [40,41,42,43,44,45]. Rab3A interconverts between a GTP-bound active state and a GDP-bound inactive state [40,46]. At ribbon synapses, Rab3A most likely plays a particularly prominent role [34,47,48]. Rab3A mediates vesicle delivery to the synaptic ribbon [34] and antibodies against Rab3 immunolabel synaptic ribbons in hair cells [48]. Binding and release of synaptic vesicles depends upon a GTP/GDP cycle [34] emphasizing the importance of proteins that regulate the nature of the guanine nucleotide bound to Rab3A. Rabconnectin-3α (RC3α)—alternative name Dmx-like 2 (DMXL2) [49]—serves, together with rabconnectin-3β, as a scaffold for the GDP/GTP exchange factor (GEF) protein and the GTPase activating protein (GAP) of Rab3A [50,51]. Therefore, we aimed to analyse the distribution of rabconnectin-3α/DMXL2 (RC3α/DMXL2), in retinal ribbon synapses and focused on photoreceptor ribbon synapses that are characterized by particularly large synaptic ribbons and intense synaptic vesicle trafficking. In the present study, we found RC3α/DMXL2 located to the synaptic ribbon in rod photoreceptor synapses of the mouse retina (a rod photoreceptor-dominated retina) using different antibodies against RC3α/DMXL2 and light and electron microscopic immunolabelling techniques suggesting the importance of proteins that regulate the nucleotide binding state of Rab3A for ribbon-associated vesicle trafficking and related events at the ribbon synapse.

## 2. Materials and Methods

### 2.1. Materials

#### 2.1.1. Mice

All mouse care/organ dissection procedures were approved by the responsible local authorities (Landesamt für Verbraucherschutz; Geschäftsbereich 4; 66115 Saarbrücken, Germany; GB 3-2.4.2.2-25-2020). Prior to organ collection, C57BL/6J mice were deeply anaesthetized with isoflurane. Mice were killed by cervical dislocation. Organ isolation was performed within 5 min *post-mortem*.

#### 2.1.2. Primary Antibodies

##### Antibodies against Rabconnectin3α/DMXL2

Two mouse monoclonal antibodies against RC3α/DMXL2 (clones 2G2 and 12D8) were generated and used in the present study for the determination of the localization of RC3α/DMXL2 in the retina. Mouse monoclonal antibodies were raised against recombinant, bacterially expressed and purified GST fusion protein in which the following 110 amino acid long peptide stretch from mouse RC3α/DMXL2 (from N- to C-terminal: KKDQLDSVSGRMENGPSESKPVSRSDGGSGADWSAVTSSQFDWSQPMVTVDEEPLRL DWGDDHDGALEEDDGGGLVMKTTDAKKAGQEQSASDPRALLTPQDEECADGDTE) was fused to the carboxy-terminus of GST using standard DNA cloning techniques. Fusion protein expression and purification, immunization, hybridoma screening, hybridoma sub-cloning, counter-screening against GST and antibody isotyping was performed by Absea (Beijing, China). Two antibody hybridoma clones against RC3α/DMXL2 were used in the present study (2G2 and 12D8; both IgG2a immunoglobulins). For peptide array experiments the antibodies were used in a 1:20,000 dilution (~10 ng/mL final immunoglobulin concentration) and for immunofluorescence (IF) and post-embedding immunogold microscopy in a 1:10 dilution (~20 μg/mL final immunoglobulin concentration).

Furthermore, a commercially available rabbit polyclonal antibody against RC3α/DMXL2 (HPA039375; Sigma) was also used in this study. The affinity-purified antibody has been raised against a recombinant protein fragment of 92 amino acids length of human RC3α/DMXL2: (from N- to C-terminal: TKTSALSAKKDQPDFISHRMDDVPSHSKALSDGNGSSGIEWSNVTSSQYDWSQPIVKVDEEPLNLDWGEDHDSA LDEEEDDAVGLVMKSTDA). This peptide sequence from human DMXL2 largely, though not completely, corresponds to the peptide region of mouse RC3α/DMXL2 that was used for the generation of the monoclonal antibodies 2G2/12D8 (see first paragraph in Section 2.1.2). The affinity-purified polyclonal antibody against RC3α/DMXL2 was used for WB in a 1:1000 dilution; for peptide array experiments in a 1:20,000; for IF in a 1:300 dilution. Further primary antibodies used in the present study have been summarized in Table 1.

#### 2.1.3. Secondary Antibodies

All secondary antibodies used in the present study have been summarized in Table 2.

#### 2.1.4. Additional Materials

Silver Enhancement Kit (Nanoprobes 2012 45ML, Nanoprobes, Inc. 95 Horseblock Road, Unit 1 Yaphank, NY, USA).

HiMark^TM^ Pre-stained protein standard (Invitrogen LC 5699).

Roti-Mark Standard for SDS-PAGE.

#### 2.1.5. Plasmids

pGEX-mouse RC3α/DMXL2 (Absea), was cloned in pGEX-KG via BamHI/XhoI using synthetic DNA encoding aa 1919–aa 2029 of mouse RC3α/DMXL2 (NP_766359) using standard methods.

### 2.2. Methods

#### 2.2.1. Embedding of Mouse Retinas and Immunohistochemistry on 0.5 μm Thin Resin Sections

Mouse retinas were processed for immunofluorescence (IF) microscopy on 0.5 μm thin resin sections as described [25,52,54,55,56,57,58,59]. Semithin sections provide a better resolution than cryostat sections for immunolabelling [60] and are capable of resolving single rod terminals and single rod active zones [52,54,56,57,58]. Eyes were isolated within 5 min *post-mortem* and the dissected posterior eyecups were flash-frozen in liquid nitrogen-cooled isopentane. Lyophilization of the tissue was performed at ~10^−7^ mbar for ~48 h. During the lyophilization, the tissue was continuously cooled by liquid nitrogen. Freeze-dried samples were equilibrated to room temperature, infiltrated with Epon resin at 28 °C on a rotor (at 2 rpm, for ~24 h), degassed in a vacuum chamber and subsequently polymerized at 60 °C for ~24 h, as described [25,52,54,55,56,57,58,59].

Semi-thin sections (0.5 µm thin) were cut from the polymerized tissue blocks with a Reichert ultramicrotome, as described [56,57]. Sections were collected on glass coverslips. Epon resin was removed from the sections by incubating sections with sodium methylate (30% *w*/*v* in methanol (Sigma-Aldrich #8.18194), 10 min); xylene/methanol (1:1 *v*/*v*, 10 min); acetone (2 × 10 min), H_2_O (10 min) and PBS (10 min), as previously described [25,52,54,55,56,57]. All resin removal steps were performed at room temperature (RT).

Next, sections were incubated simultaneously with the indicated primary antibodies overnight at 4 °C, as also previously described [25,52,55,56,57]. Following the incubation in the primary antibody dilutions, sections were washed several times with PBS and incubated with the corresponding fluorophore-conjugated secondary antibodies (1 h at RT). After several washes with PBS, immunolabelled sections were mounted with an N-propyl gallate-containing anti-fading solution, as previously described [25,52,55,56,57]. Immunolabelling experiments were performed with three different sets of embedded mouse retinas.

In control experiments, sections were incubated also without primary antibody; all other steps of the immunolabelling protocol remained the same. Additional controls were performed for double immunolabelling experiments by setting individual laser power lines to zero. The detection settings remained unchanged. These controls were performed to make sure that the immunosignals in the respective detection channel do not result from signals of the “neighbouring” detection channel (“bleed-through controls”). Immunolabelled retina sections were analysed by confocal microscopy, as described in the next paragraph.

#### 2.2.2. Confocal Microscopy of Immunolabelled Sections

We used an A1R laser scanning microscope (Nikon, Düsseldorf, Germany) for confocal microscopy, as previously described [25,52,55,56,57,61]. Images were acquired with 60×/1.40 N.A. oil objective and the 488 nm and 568 nm laser excitation lines. Image acquisition was performed with the NIS Elements software (NIS Elements AR 3.2, 64 bit; Nikon, Düsseldorf, Germany).

#### 2.2.3. Preparation and Immunolabelling of Retinal Cryostat Sections

The posterior eyecups were dissected within 5 min of *post-mortem* and flash-frozen in liquid nitrogen-cooled isopentane, as previously described [20,62]. Cryostat sections of 8 μm were cut from these samples with a Leica cryostat CM950. Cryosections were heat-fixed by putting them on a heating pad (30 min at 60 °C). Incubation of the heat-fixed cryosections with primary and secondary antibodies, negative and positive controls as well as bleed-through controls was performed as described above for semi-thin sections. Immunolabelled sections were analysed by confocal microscopy, as described above.

#### 2.2.4. Embedding of Retinas in LR Gold for Post-Embedding Immunogold Electron Microscopy

Mouse retinas were processed for post-embedding immunogold labelling as previously described [20,25,56]. Retinas, dissected as described above, were fixed overnight in 2% freshly depolymerized paraformaldehyde in PBS (pH 7.4) at 4 °C. Afterward, samples were dehydrated with ethanol (30% ethanol (4 °C, 10 min); next with ethanol concentrations of 50%, 70%, 80% to 99% ethanol (20 min each step, at −20 °C with mild agitation using an overhead rotator). Samples were infiltrated with increasing concentrations of LR Gold (ethanol/LR-Gold: 2/1, 1/1, 1/2 (*v*/*v*); 1 h each, at −20 °C) as described [20,25,56]. Samples were transferred to pure LR Gold resin (overnight at −20 °C) and finally infiltrated with LR Gold containing 0.1% benzil (*w*/*v*). Polymerization was performed for ≈48 h at −20 °C with UV light. Ultrathin sections (≈70 nm thin) were cut with a Reichert–Jung ultramicrotome and collected on 100 mesh gold grids. Please note that no OsO_4_ can be used for lipid-contrasting in post-embedding immunogold electron microscopy. Therefore, membranes, e.g., synaptic vesicle membranes, are only weakly visible.

#### 2.2.5. Post-Embedding Immunogold Labelling with Ultrasmall Immunogold Particles and Subsequent Silver Intensification

Post-embedding immunogold labelling was performed largely as previously described with some modifications [20,25,56]. Ultrathin sections were obtained from LR Gold-embedded tissue and treated with blocking buffer, containing 1% bovine serum albumin (BSA) in PBS, pH 7.4 (1 h, at RT) to saturate unspecific protein binding sites [20,25,56]. Then, sections were incubated with RC3α/DMXL2 2G2 mouse monoclonal antibody diluted 1:10 in blocking buffer (overnight, at 4 °C). Following several washes with blocking buffer, ultrathin sections were incubated with goat anti-mouse secondary antibody (1:100 dilution in blocking buffer, 90 min, at RT). The secondary antibody was conjugated to ultrasmall gold particles (~1.4 nm in diameter). After incubation with the secondary antibody, sections were washed several times with PBS and treated with 2.5% glutaraldehyde in PBS (15 min, at RT). Next sections were washed with H_2_O and the immunolabeled sections were silver-intensified according to the manufacturer’s instructions in the dark (4 min, at RT). This enhancement procedure was done to improve sensitivity. Following silver enhancement, sections were washed three times with H_2_O and contrasted with 2% uranyl acetate (in H_2_O, 10 min, at RT). As negative controls in these immunolabelling experiments, incubations in which no primary antibody was applied were used. All other steps of the immunogold labelling procedure remained identical in these negative control experiments. Immunolabelling experiments were performed with three sets of LR Gold-embedded mouse retinas. Immunolabelled ultrathin sections were analysed with a Tecnai Biotwin 12 transmission electron microscope (FEI, Eindhoven, The Netherlands) operated at 100 kV [25,56]. Images were acquired with a Megaview III digital camera (Gatan, Unterschleissheim, Germany) under the control of the iTEM acquisition software (Olympus; Hamburg, Germany).

#### 2.2.6. Peptide Arrays for Antibody Epitope Mapping

We performed epitope mapping of all three RC3α/DMXL2 antibodies applied in the present study using peptides immobilized on cellulose membranes. For antibody epitope mapping of RC3α/DMXL2 antibodies, peptides of mouse RC3α/DMXL2 covering aa 1919–aa 2029 of mouse DMXL2 (NP_766359) were analysed. Peptides with a length of 20 amino acids each (overlap of 10 amino acids) were synthesized on the membrane. Peptide synthesis was accomplished on hardened cellulose membranes using a ResPepSL-Synthesizer (Intavis Bioanalytical Instruments; Cologne, Germany) [63,64,65]. Peptide arrays were processed for epitope mapping as previously described [25,66]. The membrane was activated with methanol (1 min, at RT). Next, the membrane with the peptide arrays was briefly washed with H_2_O and incubated for 2 h with binding buffer (50 mM Tris-HCl, pH 7.5, 150 mM NaCl, 0.1% Triton X-100) with mild shaking at RT. Unspecific protein binding sites were saturated by incubating the membrane in blocking buffer (1 µM BSA in binding buffer; 1 h, at RT). Following incubation in blocking buffer, membranes were incubated with the primary antibodies that are indicated in the respective experiments (2G2 and 12D8 RC3α/DMXL2 mouse monoclonal; RC3α/DMXL2 rabbit polyclonal (Sigma-Aldrich; Taufkirchen, Germany); all in a 1:20,000 dilution in blocking buffer, overnight at 4 °C). Next, the membrane was washed 3 × 10 min with blocking buffer and incubated with goat anti-mouse antibody conjugated to horseradish peroxidase (HRP) (1:10,000 in blocking buffer) for 1 h at RT on a shaker. Antibody binding was visualized by enhanced chemiluminescence (ECL) with a ChemiDoc™ XRS Gel Doc system (Bio-Rad, Feldkirchen, Germany). After ECL detection, the locations of all peptide spots were visualized by UV illumination, as described [25,66].

### 2.3. Miscellaneous Methods

#### 2.3.1. SDS-PAGE and Western Blotting

Retinas were isolated within 5 min *post-mortem* and dissolved in 200 μL hot Laemmli buffer [25]. The samples were solubilized by homogenization by up/down pipetting in a 100 μL tip and heated at 96 °C for 10 min [25]. The protein concentration of these retina samples was determined as described [67]. Retinal lysates (~50 µg total protein per lane) were separated by 5% acrylamide SDS-PAGE. Proteins were electro-transferred to nitrocellulose membrane (Protran 0.45 µm) (at 40 V, 10 h, at 4 °C). Unspecific protein binding sites were saturated by incubation in 5% skimmed milk powder in PBS (45 min, RT) followed by overnight incubation in primary antibody (4 °C). Binding of the primary antibody was detected by the respective peroxidase-conjugated secondary antibodies and analysed by enhanced chemiluminescence (ECL) using a ChemiDoc™ XRS GelDoc system (Bio-Rad, Feldkirchen, Germany).

#### 2.3.2. Expression and Purification of GST-Tagged Fusion Proteins

BL21(DE3) bacteria were transformed with the respective pGEX plasmids by electroporation and plated on Ampicillin plates. Induction, expression with IPTG and purification of recombinant GST-tagged fusion protein was performed with standard methods as previously described [20,55,68,69].

## 3. Results

**Figure 1:**
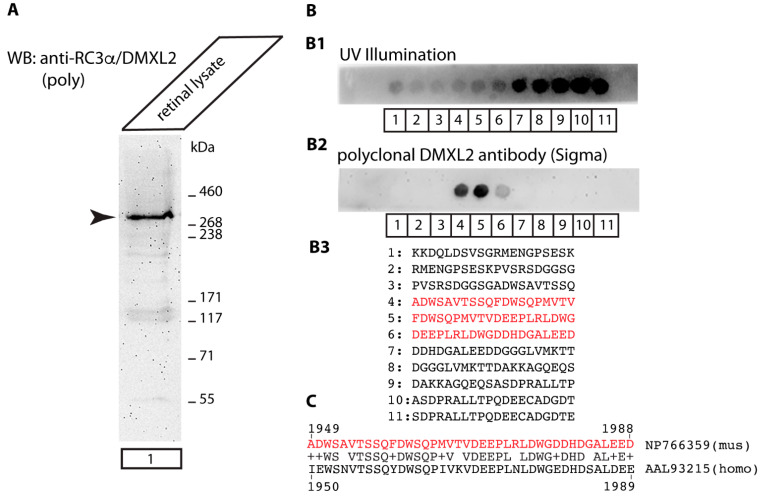
RC3α/DMXL2 expression in the mouse retina. (**A**) Retinal lysate from wild-type mice was probed by Western blot (WB) with affinity-purified rabbit polyclonal anti-RC3α/DMXL2. A high molecular weight band at ≈340 kDa was detected by the antibody in retinal lysates. (**B**) Peptide arrays that correspond to the RC3α/DMXL2 region against which this polyclonal antibody was generated, were incubated with affinity-purified rabbit polyclonal anti-RC3α/DMXL2 antibody. (**B1**) The location of all peptide spots was visualized by UV illumination. (**B2**) shows the result of the immunolabelling of the peptide array with affinity-purified rabbit polyclonal anti-RC3α/DMXL2 (ECL detection). Peptide spots #4, #5 and #6 strongly reacted with the anti-RC3α/DMXL2 polyclonal antibody. (**B3**) The amino acid sequence of spots #4, #5 and #6, that strongly reacted with the polyclonal RC3α/DMXL2 antibody, are highlighted in red. (**C**) The peptide sequence of mouse RC3α/DMXL2 (NP766359) that reacted with the affinity-purified rabbit polyclonal anti-RC3α/DMXL2 (highlighted in red) was aligned with the corresponding sequence from human RC3α/DMXL2 (AAL93215). The corresponding sequences are highly conserved between mouse and human RC3α/DMXL2 (73% amino acid identities).

We first applied a commercially available affinity-purified polyclonal rabbit antibody against DMXL2 to determine the localization of RC3α/DMXL2 in the retina. The polyclonal antibody detected a single high molecular weight band in WB analyses at the characteristic running position of ~340 kDa in mouse retinal lysates (Figure 1A). These Western blotting data clearly demonstrated that RC3α/DMXL2 is expressed in the retina. With peptide arrays that covered the entire region used for immunization we determined the precise epitopes of RC3α/DMXL2 which the polyclonal antibody reacts with (Figure 1B). The polyclonal antibody reacts with aa1949 to aa1988 of mouse RC3α/DMXL2 (NP_766359.2). This peptide region corresponds to aa1950 to aa1989 of human RC3α/DMXL2 (AAL93215) and is highly conserved between mouse and human DMXL2 (Figure 1C).

**Figure 2:**
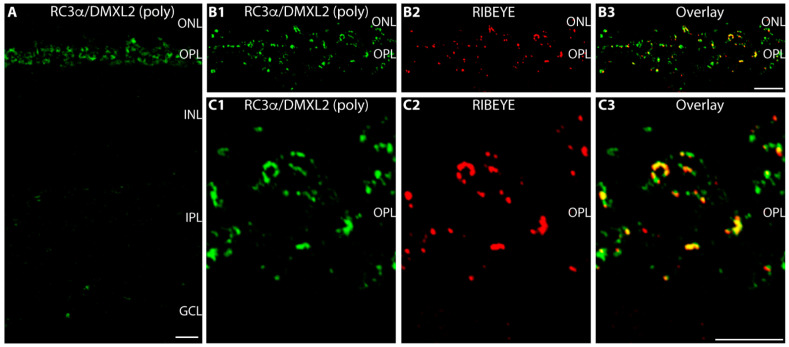
RC3α/DMXL2 is highly expressed in photoreceptor synapses in the OPL in close vicinity to synaptic ribbons. (**A**) 0.5 μm-thin retina sections incubated with affinity-purified rabbit polyclonal antibody against RC3α/DMXL2. RC3α/DMXL2 immunosignals are strongly enriched in the OPL in which photoreceptor ribbon synapses are found. (**B1**–**B3**,**C1**–**C3**) 0.5 μm-thin sections of the retina double-immunolabelled with rabbit anti-RC3α/DMXL2 (green channel) and with mouse anti-RIBEYE (2D9) (red channel). Signals from green and red channels (**B1**,**C1**/**B2**,**C2**) were overlaid in (**B3**/**C3**). Abbreviations: ONL, outer nuclear layer; OPL, outer plexiform layer; INL, inner nuclear layer; IPL, inner plexiform layer; GCL, ganglion cell layer. Scale bars: 5 μm.

Next, we used the polyclonal RC3α/DMXL2 antibody for immunolabelling of 0.5 μm thin sections obtained from mouse retina (Figure 2). In cross-sections of the retina, we observed strong RC3α/DMXL2 immunosignals in the outer plexiform layer (OPL) in which the photoreceptor ribbon synapses are located (Figure 2A). We found the inner plexiform layer only weakly, if at all, immunolabeled (Figure 2A). Therefore, we focused on the localization of RC3α/DMXL2 in photoreceptor synapses of the OPL. Photoreceptor synapses in the mouse retina are predominantly rod photoreceptors synapses [7,70]. Rod photoreceptor synapses typically possess a single, large active zone with a single and large horseshoe-shaped synaptic ribbon [7,70]. The entire presynaptic terminal is filled with many highly motile synaptic vesicles that can bind to the synaptic ribbon [71].

**Figure 3:**
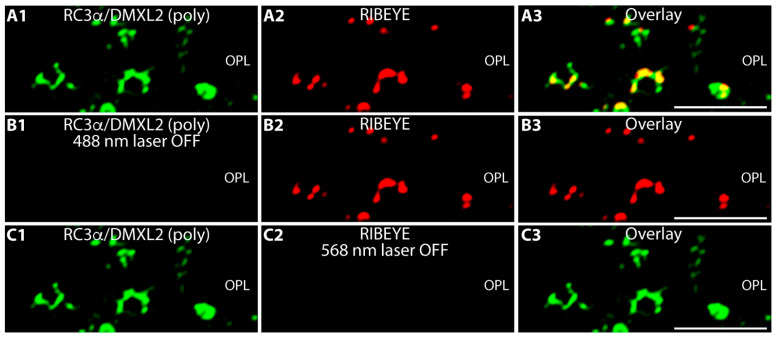
Control exposures. (**A1**–**A3**) 0.5 μm-thin retina sections were immunolabelled with rabbit anti-RC3α/DMXL2 (green channel) and with mouse anti-RIBEYE (2D9) (red channel), as in Figure 2. (**B1**–**B3**) The same immunolabelled retina section as shown in (**A1**–**A3**), but with the 488 nm laser turned off (with all PMT detection settings remaining unchanged). (**C1**–**C3**) Same double-immunolabelled retina section as shown in (**A1**–**A3**), but with the 568 nm laser turned off (with all PMT detection settings remaining unchanged). Signals from green and red channels (**A1**,**B1**,**C1**/**A2**,**B2**,**C2**) were overlaid in (**A3**,**B3**,**C3**). Abbreviations: OPL, outer plexiform layer. Scale bars: 5 μm.

Double-immunolabelling with antibodies against RIBEYE confirmed the synaptic localization of DMXL2 in the OPL (Figure 2B,C). Double immunolabelling with antibodies against RIBEYE showed partial co-localization of RC3α/DMXL2 with RIBEYE and suggested that a significant portion of RC3α/DMXL2 could be localized to the synaptic ribbon (Figure 2(B1–B3,C1–C3)). “Bleed-through” controls demonstrated that the RC3α/DMXL2 immunosignals at the synaptic ribbon are not influenced by RIBEYE immunosignals from the neighbouring detection channel but completely persist if the excitation for the RIBEYE channel is completely switched off (Figure 3). These data show that the RC3α/DMXL2 immunosignals do not result from a “bleed-through” from the RIBEYE immunosignals.

Unfortunately, the rabbit RC3α/DMXL2 antibody was not suitable for electron microscopic analyses and the ultrastructural distribution of RC3α/DMXL2 in rod photoreceptor synapses could thus not be resolved with this antibody.

**Figure 4:**
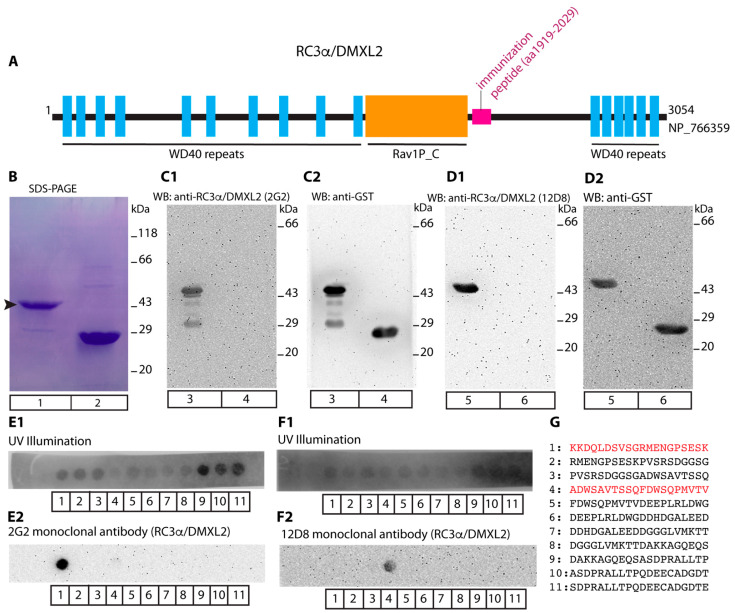
(**A**) Schematic domain structure of RC3α/DMXL2. Amino- and carboxyterminal WD40 repeats (blue boxes) and the central Rav1p_C domain (orange box) are schematically depicted. Monoclonal antibodies (2G2 and 12D8) were generated against a peptide stretch downstream of the Rav1p_C-domain (aa1919–aa2029, highlighted in pink). (**B**) SDS-PAGE of the purified GST-tagged fusion proteins (Coomassie Blue stained gel). Lane 1: RC3α/DMXL2 (aa1919–aa2029)-GST, lane 2: GST alone. (**C1**,**D1**) WB analyses of purified GST fusion proteins probed with RC3α/DMXL2 monoclonal antibodies. RC3α/DMXL2 (aa1919–aa2029)-GST was applied in lanes 3 and 5; GST in lanes 4 and 6 of (**C1**,**C2**,**D1,D2**). In (**C1**), RC3α/DMXL2-GST and GST were probed with anti- RC3α/DMXL2 (2G2). In (**C2**) the same blot was re-probed with anti-GST to verify equal loading. In (**D1**), RC3α/DMXL2 (aa1919–2029)-GST and GST were probed with anti-RC3α/DMXL2 (12D8). In (**D2**) the same blot was re-probed with anti-GST to verify equal loading. (**E1**,**E2**,**F1,F2**) show peptide arrays that cover the protein region of RC3α/DMXL2 against which the antibodies were generated (aa1919–aa2029). These peptide arrays were probed with the indicated monoclonal anti-RC3α/DMXL2 antibodies to determine the precise binding epitope of the antibodies. (**E1**,**F1**) UV light was used to visualize the location of all peptide spots. (**E2**) shows the peptide array that was incubated with monoclonal anti-RC3α/DMXL2 2G2; (**F2**) shows the peptide array incubated with monoclonal anti-RC3α/DMXL2 12D8. Peptide spot #1 (KKDQLDSVSGRMENGPSESK) strongly reacted with the 2G2 monoclonal antibody and peptide spot #4 (ADWSAVTSSQFDWSQPMVTV) with the 12D8 monoclonal antibody. (**G**) Amino acid sequences of all peptide spots. The peptide sequences of peptide spots #1 and #4, that strongly reacted with anti-RC3α/DMXL2 monoclonal antibodies 2G2 and 12D8, are highlighted in red.

In order to also resolve the ultrastructural distribution of RC3α/DMXL2 in rod photoreceptor synapses, we generated novel monoclonal antibodies against RC3α/DMXL2. Two monoclonal RC3α/DMXL2 antibodies, 2G2 and 12D8, were raised against a GST fusion protein containing a 110 amino acid long peptide stretch carboxyterminal of the central Rav1P_C domains of RC3α/DMXL2 (Figure 4A,B). Both monoclonal RC3α/DMXL2 antibodies strongly reacted with the RC3α/DMXL2-GST fusion protein in Western blot (WB) analyses (Figure 4(C1) lane 3; Figure 4(D1), lane 5), but not with GST alone (Figure 4(C1) lane 4; Figure 4(D1), lane 6). The same WB membranes that were first incubated with RC3α/DMXL2 antibodies were then re-probed with GST antibodies to analyse equal loading of the respective fusion proteins (Figure 4(C2,D2)). With peptide arrays we determined the precise binding epitopes of the RC3α/DMXL2 antibodies 2G2 and 12D8. The 2G2 antibody strongly reacted with the RC3α/DMXL2 peptide KKDQLDSVSGRMENGPSESK (Figure 4(E2),G) whereas 12D8 reacted with the RC3α/DMXL2 peptide ADWSAVTSSQFDWSQPMVTV (Figure 4(F2),G). UV illumination was used to determine the localization of the peptide spots (Figure 4(E1,F1)).

**Figure 5:**
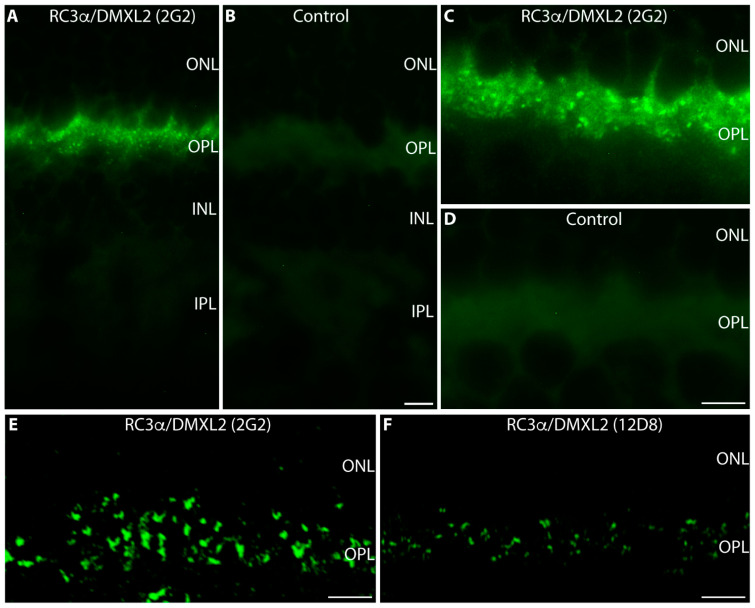
Single immunolabelling of mouse retina sections with the monoclonal RC3α/DMXL2 antibodies 2G2 and 12D8. In (**A**,**C**), 10 μm—thick cryostat sections of the mouse retina were immunolabelled with the indicated antibodies; in (**E**,**F**) 0.5 μm thin resin sections of the mouse retina. (**B**,**D**) represent control incubations in which the primary antibody was omitted. All other steps of the immunolabelling procedure were identical. Abbreviations: ONL, outer nuclear layer; OPL, outer plexiform layer; INL, inner nuclear layer; IPL, inner plexiform layer. Scale bars: 5 μm.

Both novel monoclonal RC3α/DMXL2 antibodies 2G2 and 12D8 confirmed the previously observed synaptic localization of RC3α/DMXL2 (Figure 5) that has been obtained with the affinity-purified, rabbit polyclonal RC3α/DMXL2 antibody (Figure 2 and Figure 3). Both antibodies (2G2 and 12D8) generated strong RC3α/DMXL2 immunosignals in the OPL (Figure 5A,C,E,F). The RC3α/DMXL2 antibodies 2G2 and 12D8 worked both on cryostat sections (Figure 5A,C) and on semi-thin sections (Figure 5E,F). The resolution of semi-thin sections was better than the resolution of cryostat sections (Figure 5A,C vs. Figure 5E,F).

The 2G2 and 12D8 RC3α/DMXL2 mouse monoclonal antibodies produced discrete, punctate immunosignals in the OPL, that partly appeared horseshoe-shaped (Figure 5A,C,E,F). The 2G2 antibody generated stronger immunosignals in the OPL and thus appeared more suitable for further immunocytochemical analyses.

**Figure 6:**
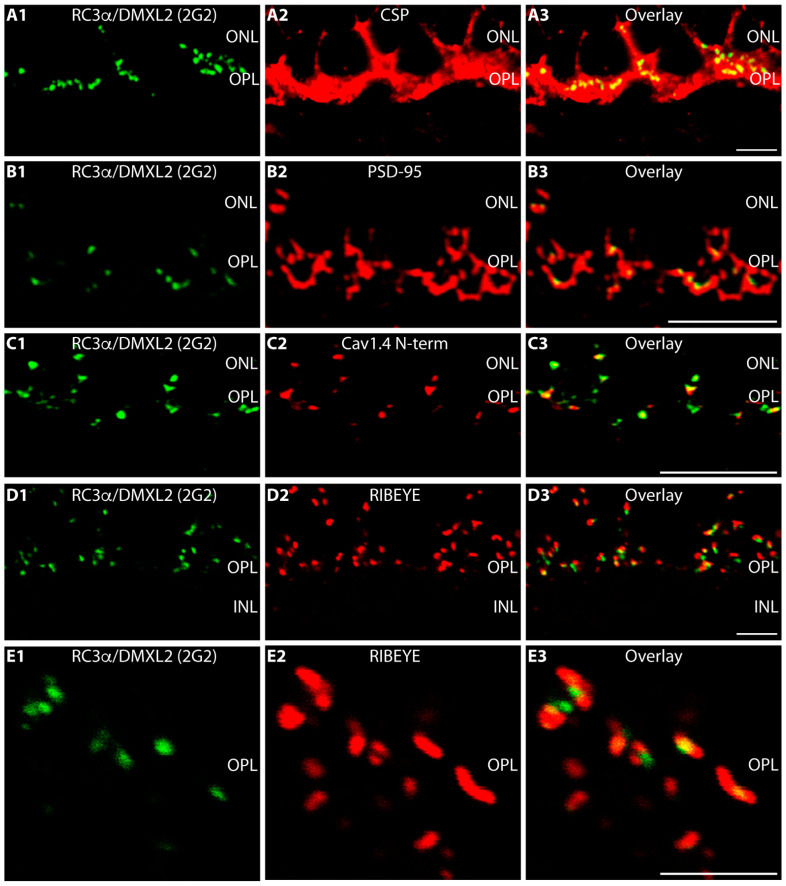
RC3α/DMXL2 is strongly expressed in photoreceptor synapses of the OPL in close vicinity to the synaptic ribbons. (**A1**–**A3**,**B1**–**B3**,**C1**–**C3**,**D1**–**D3**,**E1**–**E3**) Retina sections double-immunolabelled with mouse anti-RC3α/DMXL2 2G2 (**A1**,**B1**,**C1**,**D1**,**E1**) and with rabbit antibodies against CSP (**A2**), PSD-95 (**B2**), Cav1.4 (**C2**) and RIBEYE (**D2**,**E2**). Signals from green channels (**A1**,**B1**,**C1**,**D1**,**E1**) and red channels (**A2**,**B2**,**C2**,**D2**,**E2**) were overlaid in (**A3**,**B3**,**C3**,**D3**,**E3**). In (**A1**–**A3**,**D1**–**D3**,**E1**–**E3**), 10 μm-thick cryostat sections were used for immunolabelling; in (**B1**–**B3**,**C1**–**C3**) 0.5 μm—thin resin sections. Abbreviations: ONL, outer nuclear layer; OPL, outer plexiform layer. Scale bars: 5 μm.

The RC3α/DMXL2 (2G2) immunosignals were located within the photoreceptor presynaptic terminals as judged by double-immunolabelling with antibodies against cysteine-string protein (CSP), a synaptic vesicle protein of the presynaptic photoreceptor terminal (Figure 6(A1–A3)). As mentioned above, the entire large presynaptic photoreceptor terminal is occupied by many synaptic vesicles that contain CSP [72]. Double-immunolabelling with anti-PSD-95 revealed that RC3α/DMXL2 is located within the presynaptic terminal close to the presynaptic plasma membrane (Figure 6(B1–B3)). Please note that PSD-95 (postsynaptic density protein-95) is a presynaptic protein in photoreceptor synapses [73]. In photoreceptor synapses, PSD-95 is located beneath the presynaptic plasma membrane of the presynaptic terminal [73]. Immunosignals for RC3α/DMXL2 and voltage-gated Cav1.4 calcium channels, which are located at the presynaptic active zone close to the synaptic ribbon [58,74], showed a similar localization/distribution at the light microscopic level (Figure 6(C1–C3)). Double-immunolabelling with antibodies against RIBEYE further indicated an enrichment of RC3α/DMXL2 at the synaptic ribbons (Figure 6(D1–D3,E1–E3)).

**Figure 7:**
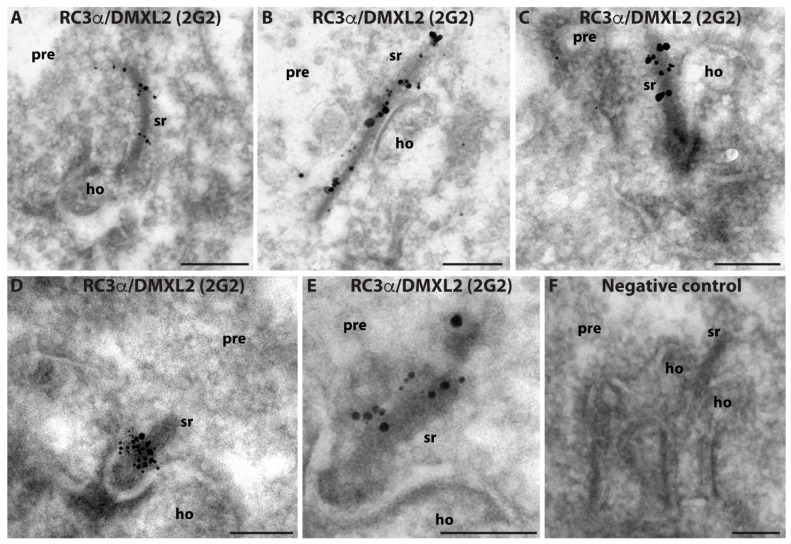
Post-embedding immunogold labelling of ultrathin LR Gold sections from the mouse retina (**A**–**F**). Rod photoreceptor synapses were immunolabelled with monoclonal anti-RC3α/DMXL2 2G2 antibody (**A**–**E**). (**F**) shows a representative negative control incubation in which the primary antibody was omitted. All other steps of the immunolabelling procedure remained the same. Please note that membrane contrast of membranes is limited because a post-embedding approach (without usage of OsO_4_) was applied. Secondary goat anti-mouse antibodies were conjugated to ultrasmall (1.4 nm diameter) gold particles that were subsequently silver-intensified. Abbreviations: sr, synaptic ribbon; pre, presynaptic; ho, postsynaptic dendrites of horizontal cells. Scale bars: 300 nm (**A**–**F**).

The RC3α/DMXL2 2G2 mouse monoclonal antibody against RC3α/DMXL2 was suitable for post-embedding electron microscopic immunolabelling analyses (Figure 7). Post-embedding immunogold electron microscopy demonstrated that the synaptic ribbon is strongly decorated by the RC3α/DMXL2 (2G2) antibody. These data demonstrate also at the ultrastructural level that the synaptic ribbon is associated with RC3α/DMXL2, confirming the light microscopic immunolabelling data obtained with the polyclonal and monoclonal RC3α/DMXL2 antibodies. Other components of the presynaptic terminal were not strongly immunolabelled in our post-embedding immunogold labelling approach.

## 4. Discussion

In the present study we have shown that rabconnectin3α (RC3α)/DMXL2 is localized to the synaptic ribbon in rod photoreceptor synapses of the mouse retina. The presence of RC3α/DMXL2 at the synaptic ribbon was consistently shown with three independent RC3α/DMXL2 antibodies at the light microscopic level using high resolution confocal microscopy. The localization at the synaptic ribbon was also confirmed at the ultrastructural level. Post-embedding immunogold electron microscopy demonstrated the presence of RC3α/DMXL2 at the synaptic ribbon in rod photoreceptor synapses. The retina of mice is a rod-dominated retina; more than 95% of photoreceptor synapses are made by rod photoreceptors [7]. Rod synapses have a very characteristic morphology at the light- and electron microscopic level [7]. Whether cone synaptic ribbons are also associated with RC3a/DMXL2 remains to be shown by future investigations. In the inner plexiform layer, we did not observe an obvious RC3α/DMXL2 immunosignal which might be based on the much smaller size of synaptic ribbons in the IPL in comparison to the OPL [7]. Thus, RC3α/DMXL2 immunosignals in the IPL might be under the detection limit for immunofluorescence microscopy on semi-thin sections.

Based on its subcellular localization at rod photoreceptor synaptic ribbons identified in the present study, RC3α/DMXL2 might serve as an acceptor complex for synaptic vesicles at the synaptic ribbon. As mentioned, RC3α/DMXL2 serves as a scaffold that binds GAP and GEF proteins that interact with Rab3A and determine the nature of its bound nucleotide (GDP vs. GTP) and the activity status. Rab3A is a component of synaptic vesicles, also at retinal ribbon synapses [75]. Thus, the previously observed binding of Rab3-containing synaptic vesicles to the synaptic ribbon [34,48] could be mediated by Rab3A interacting proteins, such as Rab3GEF/GAP, that are recruited via RC3α/DMXL2 to the synaptic ribbon. Electron-dense connections (“tethers”) between synaptic vesicles and synaptic ribbons have been identified by electron microscopy [76]. Rab3A and Rab3A effectors have been previously proposed to be components of these tethers [34,48]. The known association of RC3α/DMXL2 with both GAP and GEF proteins of Rab3A [50,51] suggests that GTP/GDP exchange processes of Rab3A occur at the synaptic ribbon. Rab3A interacts with important Rab3A effectors in a GTP/GDP-dependent manner [40,46,47]. Interestingly, GTP/GDP-dependent interactors of Rab3A are enriched at the synaptic ribbon complex [20,47,74,75,77,78]. These include the RIM family of active zone proteins, which are important effectors of depolarization-evoked synaptic vesicle fusion at photoreceptor ribbon synapses [47,77]. Therefore, these GTP/GDP exchange processes will likely be functionally relevant for the intense vesicle trafficking events associated with the synaptic ribbon.

In the brain, RC3α/DMXL2 was purified from a crude synaptic vesicle fraction [50,51,79] and RC3α/DMXL2 has been localized to synaptic vesicles in the brain by a pre-embedding immunolabelling approach [50]. In the mouse retina, we did not find a RC3α/DMXL2 signal on most of the synaptic vesicles present in rod photoreceptor presynaptic terminals. We do not want to exclude that RC3α/DMXL2 is also present on non-ribbon-associated synaptic vesicles in ribbon synapses. The epitopes of RC3α/DMXL2 might be blocked/inaccessible on synaptic vesicles in post-embedding immunogold labelling procedures or the amount of RC3α/DMXL2 on vesicles in ribbon synapses could be less compared to the amount of RC3α/DMXL2 at the synaptic ribbon and too low to be detected by our antibodies. Further investigations are needed to analyse these possibilities.

RC3α/DMXL2 is a large protein with multiple amino- and carboxyterminal WD40 repeats and a central Rav1P_C domain. Interestingly, RC3α/DMXL2 not only interacts with GAP/GEF proteins of Rab3 but also with vesicular protein H^+^-ATPases [80,81,82,83]. RC3α/DMXL2 is homologous to the yeast Rav1 protein, a central component of the yeast RAVE (Regulator of H^+^-ATPase of vacuolar and endosomal membranes) complex [83,84,85]. The rabconnectin3 complex (RC3α/DMXL2 and RC3β) in higher eucaryotes/RAVE complex in yeast was shown to interact with components of vesicular H^+^-ATPases [82,83,84,86,87]. In hair cells of zebrafish, RC3α/DMXL2 promotes the assembly of the vesicular H^+^-ATPase and its functional activity that results in the acidification of synaptic vesicles [88]. RC3α/DMXL2 has been localized by immunofluorescence microscopy to the basal portion of hair cells [88]. At this location, ribbon synapses are found in hair cells [88].

Acidification of synaptic vesicles and a proton electrochemical gradient generated by the activity of the vesicular H^+^-ATPase drive the loading of neurotransmitter into the synaptic vesicles (for review, [89,90]). Full acidification allows complete neurotransmitter loading of the synaptic vesicles, which was shown to be relevant for synaptic signalling [89,90,91]. Furthermore, full acidification of the synaptic vesicle lumen leads to the dissociation of V_o_/V_1_ complexes of the vesicular H^+^-ATPase and to a change (increase) in fusion competence of the respective vesicles [91]. Of note, the vesicular H^+^-ATPase has been found in protein complexes that were immunopurified with RIBEYE antibodies [48]. Thus, the association of RC3α/DMXL2 with the synaptic ribbon might ensure that only fully signalling-competent synaptic vesicles, i.e., synaptic vesicles that are completely filled with neurotransmitter, will be made available to the active zone. The association of RC3α/DMXL2 with the synaptic ribbon could help to prevent synaptic transmission failures that might result from the fusion of synaptic vesicles that are not or incompletely filled with neurotransmitter. To prevent failures in synaptic transmission seems particularly important for rod synapses that can faithfully transmit even very weak signals, e.g., tiny membrane potential changes caused by the absorption of a single photon. A proton electrochemical gradient-dependent loading with glutamate has been identified as an important determinant of synaptic vesicle quantal size (amount of neurotransmitter in a synaptic vesicle) [92,93].

Interestingly, the rabconnectin3 complex, consisting of RC3α/DMXL2 and RC3β, has also been reported to interact with CAPS1 (via rabconnectin-3β; [94]). CAPS1 has been characterized in the brain as a synaptic protein with a dual role in vesicle priming and neurotransmitter filling. The localization and function of CAPS1 in the retina remains to be elucidated. Furthermore, RC3α/DMXL2 was found to interact with voltage-gated Cav-channels in conventional synapses [95]. These data propose a central role of RC3α/DMXL2 for synaptic signalling.

The association of RC3α/DMXL2 with human diseases, e.g., Ohtahara syndrome (a syndromic deafness-associated disease with mutations in the RC3α/DMXL2 gene) and some non-syndromic hearing losses with sensorineural impairment [96,97,98], emphasizes the importance to further explore its function at ribbon synapses, including ribbon synapses of the retina.

## Figures and Tables

**Table 1:** Further primary antibodies.

Antibody	Source	Reference	Dilution
RIBEYE(B) U2656, rabbit polyclonal	Lab-made	[20]	1:10,000 (IF)
RIBEYE(B) (2D9), mouse monoclonal	Lab-made	[52]	1:200 (IF) 1:400 (EM)
PSD-95 (postsynaptic density protein-95), rabbit polyclonal	Gift Dr. T.C. Südhof	[53]	1:1000 (IF)
CSP (cysteine-string protein), rabbit polyclonal	Lab-made	Raised against recombinant full-length mouse CSP	1:500 (IF)
Cav1.4 Nterm, rabbit polyclonal	Lab-made	[54]	1:500 (IF)
GST, mouse monoclonal	Sigma-Aldrich, G1160	[55]	1:10,000 (WB)

**Table 2:** **Secondary** **Antibodies**.

Antibody	Source	Dilution
Donkey anti-mouse Alexa 488	Invitrogen; Karlsruhe, Germany; A-21202	1:1000 (IF)
Chicken anti-mouse DyLight 488	Jackson ImmunoResearch; 715485150	1:1000 (IF)
Chicken anti-rabbit Alexa 488	Invitrogen; Karlsruhe, Germany; A-21441	1:1000 (IF)
Chicken anti-rabbit Alexa 568	Invitrogen; Karlsruhe, Germany; A-10042	1:1000 (IF)
Chicken anti-mouse Alexa 488	ThermoFisher; Karlsruhe, Germany; 10114192	1:1000 (IF)
Goat anti-mouse peroxidase-conjugate (POX)	Sigma; Taufkirchen, Germany; A3673	1:5000 (WB)
Goat anti-mouse conjugated to 1.4 nm Nanogold	Nanoprobes/Biotrend, Cologne, Germany, #N-2001	1:100 (EM)

**Abbreviations**: Immunofluorescence (IF), Western blot (WB) with enhanced chemiluminescence (ECL) detection, Electron microscopy (EM).

## Data Availability

All data are presented in the main manuscript and the manuscript figures.
